# Molecular Differentiation of Intact West Nile Virus Using a PMAxx™-Enabled Digital PCR Workflow

**DOI:** 10.3390/ijms27094004

**Published:** 2026-04-29

**Authors:** Giuseppe Sberna, Francesca Colavita, Cosmina Mija, Fabiano Brillo, Fabrizio Carletti, Silvia Cammisa, Flavia Smoquina, Fabrizio Maggi

**Affiliations:** National Institute for Infectious Diseases Lazzaro Spallanzani—IRCCS, 00149 Rome, Italy; giuseppe.sberna@inmi.it (G.S.); mijacosmina@gmail.com (C.M.); fabiano.brillo@inmi.it (F.B.); fabrizio.carletti@inmi.it (F.C.); silvia.cammisa@inmi.it (S.C.); flavia.smoquina@inmi.it (F.S.); fabrizio.maggi@inmi.it (F.M.)

**Keywords:** West Nile virus, propidium monoazide, PMA, PMAxx, digital PCR, dPCR, virus integrity, virus infectivity

## Abstract

West Nile virus (WNV) diagnosis relies on nucleic acid amplifications, but these techniques do not discriminate between infectious and non-infectious viral particles. This limitation can be bypassed by using a genome-binding dye (PMAxx) that is unable to cross membranes and can only bind to the genomes of non-intact (i.e., non-infectious) viral particles. This study evaluated a workflow combining PMAxx treatment with digital PCR to improve the molecular discrimination of intact WNV particles. Fifty-five samples (35 plasma, 20 urine) from 41 patients with WNV fever (WNF) or WNV neuroinvasive disease (WNND) were analyzed. Samples were tested with/without PMAxx treatment. Overall, PMAxx treatment resulted in a significant reduction in detectable viral RNA (median reduction: 1.0 Log copies/mL; *p* < 0.0001), indicating that a substantial fraction of RNA detected by standard methods originated from non-infectious particles. This reduction was more visible in urine (1.8 Log copies/mL) than in plasma (0.4 Log copies/mL), suggesting a higher proportion of degraded viral particles or free RNA in urine. Stratification by clinical presentation showed significant reductions in both WNF and WNND patients, with no significant differences between groups. This approach may represent a valuable adjunct for improving diagnostic interpretation and epidemiological assessment of WNV infection, particularly in matrices characterized by prolonged RNA persistence.

## 1. Introduction

West Nile virus (WNV) is a positive-sense single-stranded RNA arbovirus belonging to Flavivirus genus in the Flaviviridae family [1]. Transmitted mainly through the bite of mosquitoes of the Culex genus, WNV is responsible for endemic and epidemic infections in many areas of the world, including Europe, Africa, the Middle East, and the Americas [2]. Although most human infections are asymptomatic, approximately 20% of individuals develop a flu-like febrile syndrome (WNV fever, WNF), and less than 1% progress to severe neuroinvasive forms (WNV neuroinvasive disease, WNND), such as meningitis, encephalitis, or acute flaccid paralysis, associated with high mortality and long-term neurological sequelae [3]. Between July and August 2025, the first outbreak of human cases of autochthonous WNV infection was reported in Italy (Lazio region), including both febrile forms and neuroinvasive manifestations [4]. This epidemic event highlighted intense and early viral circulation, with a significant impact on regional health services and epidemiological surveillance. In this context, molecular diagnosis played a central role in the identification of acute cases. Currently, laboratory diagnosis of WNV infection is based primarily on serological tests and molecular methods. Nucleic acid amplification techniques (NAATs), such as real-time RT-PCR, are a fundamental tool in the early stages of infection and in immunocompromised individuals [5]. However, an intrinsic limitation of conventional molecular methods is their inability to discriminate between viral RNA from infectious particles and residual RNA from inactivated, degraded, or no longer replicating viruses [6]. This can lead to an overestimation of the infectious viral load and complicate the clinical and epidemiological interpretation of the results.

Furthermore, it is known that the distribution and persistence of WNV vary depending on the biological matrix analyzed. Numerous studies have shown that WNV RNA can be detected in urine at higher concentrations and for a longer period than in plasma or serum, probably due to viral replication in the kidneys and the greater stability of RNA in this matrix [7,8]. These differences may also vary depending on the clinical severity of the infection, as patients with WNND may have a different viral distribution and persistence than patients with WNF. These differences suggest that the structure of viral particles and the integrity of the envelope may vary between different sample types.

Considering this evidence, there is a clear need for advanced molecular approaches that can overcome the limitations of conventional methods and provide more reliable information on the actual infectivity of the virus, especially in biological matrices characterized by prolonged persistence of viral RNA. In this context, particular interest has been focused on the development of strategies that allow discrimination between infectious and non-infectious viral particles, improving the interpretation of molecular results and their clinical and epidemiological relevance. In fact, some approaches are based on the use of photoactivatable dyes, such as propidium monoazide (PMA) and its newer version PMAxx™ [9]. PMAxx™ can selectively penetrate viral particles with damaged envelopes or capsids; once photoactivated, it binds covalently to genomes, preventing their amplification during PCR [10,11,12,13]. This approach has been successfully applied to several RNA viruses, including enteroviruses, noroviruses, and SARS-CoV-2, demonstrating a higher correlation with viral infectivity than standard molecular methods [13,14,15,16]. The combination of PMAxx™ with digital PCR (dPCR), a highly sensitive and accurate technology, allows for absolute and precise quantification of viral nucleic acids by dividing the reaction into thousands of independent microdroplets [17]. Compared to real-time quantitative PCR, dPCR offers greater robustness against reaction inhibitors and better reproducibility, making it particularly suitable for the analysis of complex biological samples [18].

The aims of this study are to assess the ability of PMAxx™ to enhance the molecular discrimination of intact WNV particles using a dPCR-based workflow and to determine whether PMAxx™ performance varies according to the biological matrix by comparing plasma and urine samples and clinical presentation, by evaluating differences between patients with WNF and those with WNND.

## 2. Results

### 2.1. Assessment of PMAxx Treatment Efficacy

**Figure 1:**
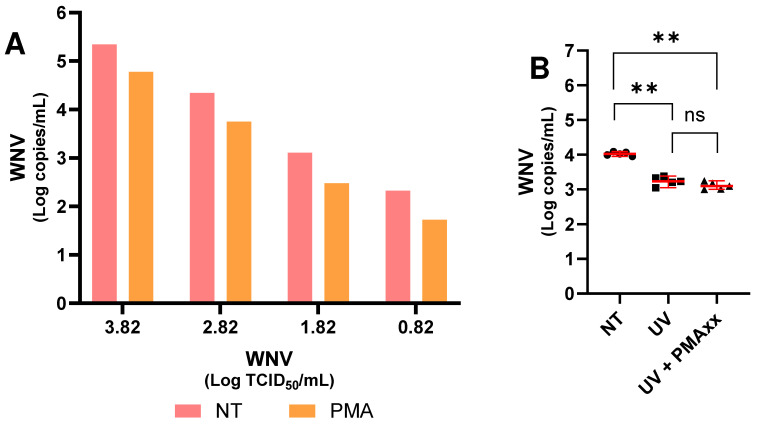
(**A**) WNV RNA levels without (NT) and with PMAxx™ treatment in serial dilutions of WNV in culture medium without fetal bovine serum (FBS). (**B**) WNV RNA levels of samples without PMAxx™ (NT), inactivated with UV light (UV), and both inactivated with UV light and treated with PMAxx™ (UV + PMAxx). Red lines indicate the median and 95% of confidence intervals; asterisks indicate statistical significance levels (** *p* < 0.01; ns: not significant).

To evaluate the performance of PMAxx against WNV, serial virus dilutions (from 10^3.82^ TCID_50_/mL to 10^0.82^ TCID_50_/mL) were prepared, allowing assessment of the effect of PMAxx (Figure 1A). An overall mean reduction of 74.6% was observed across all dilutions.

In addition, to investigate the mechanism of action of PMAxx, virus inactivation assays were performed using UV light, as this method is known to damage viral genomes without affecting the structure of viral particles. As shown in Figure 1B, the untreated virus dilution at 10^2.8^ TCID_50_/mL differed significantly in viral RNA levels from both the UV-treated samples and the samples treated with UV and PMAxx. In contrast, no significant differences were observed between the results obtained from UV-inactivated virus and those from virus inactivated by UV and subsequently treated with PMAxx (Figure 1B).

**Figure 2:**
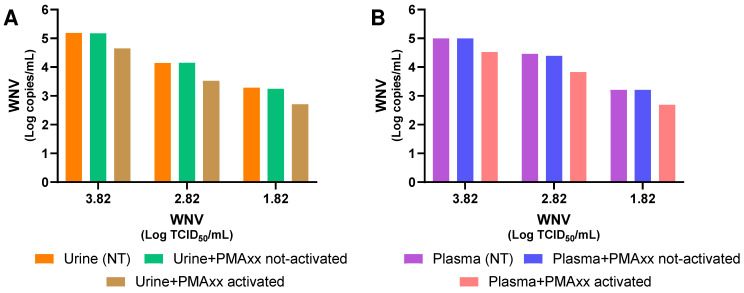
(**A**) WNV RNA levels in urine matrix, spiked with WNV, without PMAxx™ treatment (NT), treated with non-activated PMAxx, and treated with activated PMAxx. (**B**) WNV RNA levels in plasma matrix, spiked with WNV, without PMAxx™ treatment (NT), treated with non-activated PMAxx, and treated with activated PMAxx.

Finally, it was assessed whether the type of matrix in which the virus is present could influence the effect of PMAxx. For this purpose, serial dilutions of WNV (from 10^3.82^ TCID_50_/mL to 10^1.82^ TCID_50_/mL) were prepared directly in urine (Figure 2A) and in plasma matrices (Figure 2B). In the former case, an average reduction of 73.5% was observed, while in the latter, an average reduction of 71.0% was obtained.

Moreover, the same viral dilutions were used to assess whether non-activated PMAxx (which must be activated by a specific lamp (the PMA-Lite™ 2.0 LED Photolysis Device) in order to bind viral genomes) exerted any effect. As shown in Figure 2, non-activated PMAxx had no effect.

### 2.2. Effect of PMAxx Treatment on Clinical Specimens

**Figure 3:**
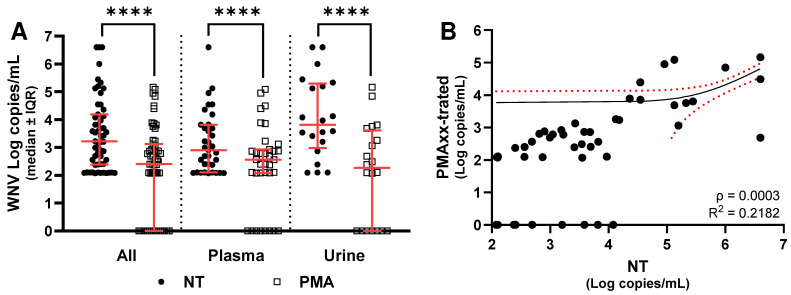
(**A**) WNV RNA levels without (NT) and with PMAxx™ treatment in all samples, plasma, and urine analyzed by dPCR. (**B**) Linear regression analysis performed between NT and the difference (Δ) between NT and PMAxx-treated, considering both plasma and urine specimens. Red lines indicate the median and interquartile range (IQR); asterisks indicate statistical significance levels (**** *p* < 0.0001). Red dot lines indicate the 95% confidence interval for the mean.

As shown in Figure 3, PMAxx treatment led to a statistically significant reduction in detectable viral RNA copies compared with untreated samples (*p* < 0.0001). This decrease was evident when considering all samples together (Figure 3A), as well as when analyzing plasma (Figure 3A) and urine samples separately (Figure 3A). When considering all 55 samples together, untreated specimens showed a median value of 3.2 Log copies/mL (IQR: 1.8 Log copies/mL), whereas PMAxx-treated samples displayed a median of 2.4 Log copies/mL (IQR: 3.1 Log copies/mL), corresponding to a median reduction of 1.0 Log copies/mL (IQR: 1.8 Log copies/mL; Figure 3A). In plasma samples, the median viral load was 2.9 Log copies/mL (IQR: 1.5 Log copies/mL), decreasing to 2.6 Log copies/mL (IQR: 0.8 Log copies/mL) after PMAxx treatment. This corresponds to a median reduction of 0.4 Log copies/mL (IQR: 1.7 Log copies/mL; Figure 3A). In urine samples, the median viral load was 3.8 Log copies/mL (IQR: 2.1 Log copies/mL), and it decreased to 2.3 Log copies/mL (IQR: 3.4 Log copies/mL) after treatment, yielding a median difference of 1.8 Log copies/mL (IQR: 0.9 Log copies/mL; Figure 3A).

**Figure 4:**
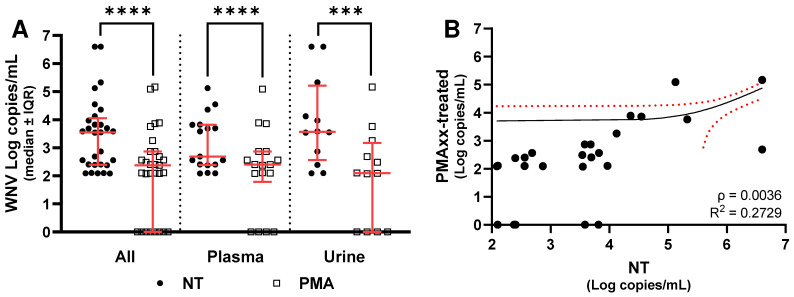
(**A**) WNV RNA levels without (NT) and with PMAxx™ treatment in all WNF samples, WNF plasma, and WNF urine samples analyzed by dPCR. (**B**) Linear regression analysis performed between NT WNF and the difference (Δ) between NT WNF and PMAxx-treated WNF, considering both plasma and urine specimens. Red lines indicate the median and interquartile range (IQR); asterisks indicate statistical significance levels (*** *p* < 0.001; **** *p* < 0.0001). Red dot lines indicate the 95% confidence interval for the mean.

When stratified by clinical presentation, both patients with WNF and those with WNND showed a significant decrease in viral RNA copies following PMAxx treatment. Among WNF patients, considering all sample types combined, untreated specimens exhibited a median viral load of 3.5 Log copies/mL (IQR: 1.6 Log copies/mL), whereas PMAxx-treated samples showed a median of 2.4 Log copies/mL (IQR: 2.9 Log copies/mL), corresponding to a median reduction of 1.3 Log copies/mL (IQR: 1.6 Log copies/mL; Figure 4A). In plasma, untreated samples had a median of 2.7 Log copies/mL (IQR: 1.4 Log copies/mL), decreasing to 2.4 Log copies/mL (IQR: 0.8 Log copies/mL) after treatment, with a median difference of 0.7 Log copies/mL (IQR: 1.2 Log copies/mL; Figure 4A). In urine samples, untreated specimens showed a median of 3.6 Log copies/mL (IQR: 2.0 Log copies/mL), while PMAxx-treated samples had a median of 2.1 Log copies/mL (IQR: 2.9 Log copies/mL), yielding a median reduction of 1.7 Log copies/mL (IQR: 0.9 Log copies/mL; Figure 4A).

**Figure 5:**
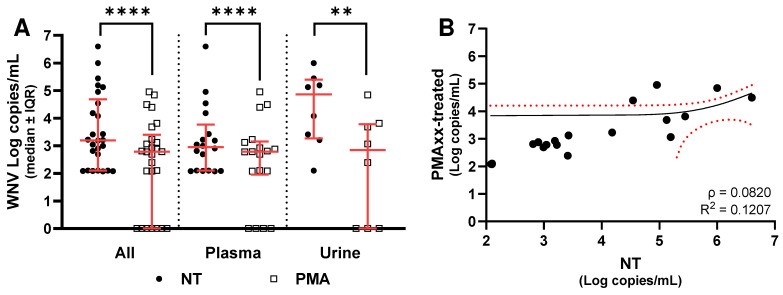
(**A**) WNV RNA levels without (NT) and with PMAxx™ treatment in all WNND samples, WNND plasma, and WNND urine samples analyzed by dPCR. (**B**) Linear regression analysis performed between NT WNND and the difference (Δ) between NT WNND and PMAxx-treated WNND, considering both plasma and urine specimens. Red lines indicate the median and interquartile range (IQR); asterisks indicate statistical significance levels (** *p* < 0.01; **** *p* < 0.0001). Red dot lines indicate the 95% confidence interval for the mean.

Among patients with WNND, when all sample types were considered together, untreated samples showed a median viral load of 3.2 Log copies/mL (IQR: 2.1 Log copies/mL), whereas PMAxx-treated samples had a median of 2.8 Log copies/mL (IQR: 1.7 Log copies/mL), corresponding to a median difference of 1.0 Log copies/mL (IQR: 1.9 Log copies/mL; Figure 5A). In plasma samples, untreated samples had a median of 2.9 Log copies/mL (IQR: 1.3 Log copies/mL), while PMAxx-treated samples showed a median of 2.8 Log copies/mL (IQR: 1.0 Log copies/mL), resulting in a median difference of 0.3 Log copies/mL (IQR: 1.8 Log copies/mL; Figure 5A). In urine samples, untreated samples had a median of 4.9 Log copies/mL (IQR: 1.9 Log copies/mL), whereas PMAxx-treated samples had a median of 2.9 Log copies/mL (IQR: 3.7 Log copies/mL), corresponding to a median difference of 1.9 Log copies/mL (IQR: 1.0 Log copies/mL; Figure 5A).

**Figure 6:**
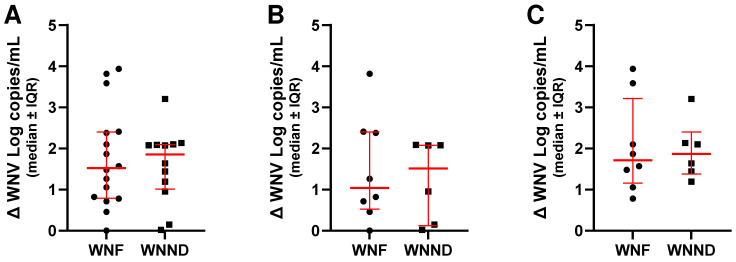
Comparison of the Δ (difference in WNV load between untreated and PMAxx treated samples) collected from the same patient at the same time point and stratified by clinical presentation (WNF: eight patients; WNND: six patients): all sample types (**A**), plasma (**B**), and urine (**C**). Red lines indicate the median and interquartile range (IQR).

Moreover, Spearman correlation test confirmed a significant association between NT values and the results of PMAxx treatment in all the three groups (*p* < 0.0001), but linear regression analysis showed a significant association when all samples were considered together (Figure 3B) or for WNF (Figure 4B); no significant association was found for WNND (Figure 5B). Finally, comparison of the median of NT−PMAxx difference (Δ) values between the two groups did not reveal any statistically significant differences in viral load reduction following PMAxx treatment, even when samples were further stratified by matrix type (Figure 6).

**Figure 7:**
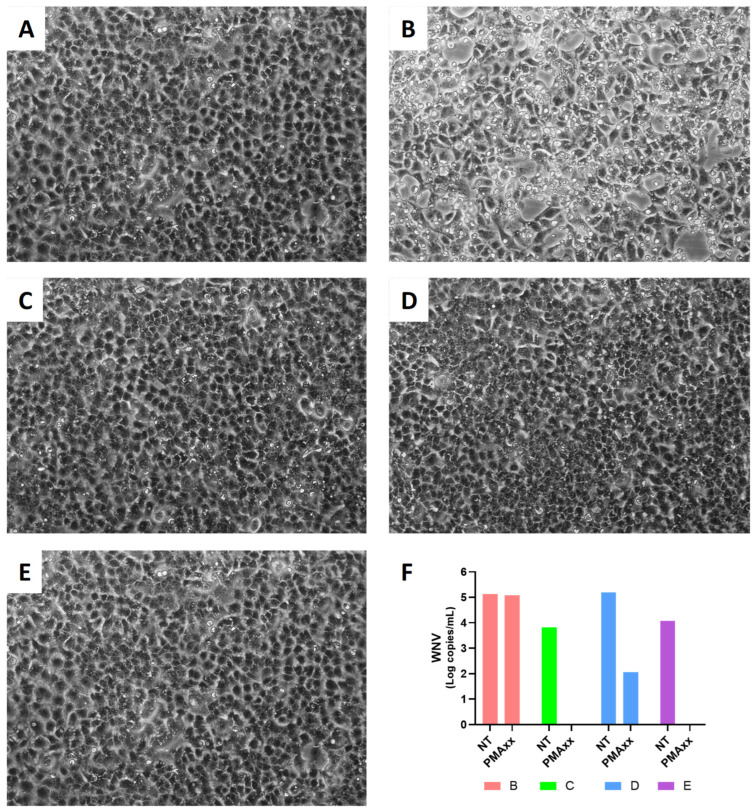
WNV viral isolations of two plasma samples, two urine specimens, and relative WNV Log copies/mL not treated (NT) and treated with PMAxx analyzed with ddPCR. (**A**) Vero E6 cell culture used as a negative control; (**B**,**C**) Vero E6 cells cultured with two plasma samples; (**D**,**E**) Vero E6 cells cultured with two urine specimens; (**F**) samples results not treated (NT) and treated with PMAxx analyzed with ddPCR. Magnification 10×.

Finally, to assess whether the results obtained after PMAxx treatment were in agreement with viral isolation (i.e., therefore, whether after treatment residual WNV particles remained intact and capable of producing a new cycle of infection), four samples (two urine and two plasma) were inoculated and cultured on VERO E6 cells (Figure 7).

In Figure 7B, the plasma sample that, when untreated, showed a WNV load of 5.1 log copies/mL and still exhibited 5.1 log copies/mL after PMAxx treatment was successfully isolated. In contrast, the other clinical samples, a plasma sample (Figure 7C) and a urine sample (Figure 7E), which tested negative for WNV after PMAxx treatment, were not isolated. The other urine sample was also not isolated; this sample showed 5.2 log copies/mL when untreated, whereas after PMAxx treatment, only 115 copies/mL were detected.

## 3. Discussion

The increasing sensitivity of molecular diagnostic techniques has profoundly improved the detection of viral infections, allowing the identification of very low amounts of viral nucleic acids in a wide range of clinical specimens [19,20]. At the same time, these technologies have a conceptual limitation: the detection of viral RNA does not necessarily reflect the presence of infectious virus [21,22]. This issue is particularly relevant for WNV, a pathogen characterized by prolonged persistence of viral RNA in specific biological compartments [23], even in the absence of cultivable virus. Moreover, although viral culture remains the reference method for assessing infectivity, its biosafety requirements and long turnaround times limit its routine use [24]. In this context, the PMAxx™-dPCR approach emerges as a practical surrogate tool that can enhance the clinical and epidemiological interpretation of WNV results.

The present study addresses a clinically and epidemiologically relevant challenge by evaluating a molecular workflow designed to improve the discrimination between RNA associated with potentially infectious WNV particles and residual RNA originating from damaged or non-replicative virions. So, it was demonstrated that the application of a PMAxx™-mediated dPCR workflow significantly improves the molecular discrimination of WNV RNA associated with structurally intact versus compromised viral particles in clinical specimens. The observed reduction in detectable viral RNA following PMAxx™ treatment across all samples suggests that a relevant proportion of WNV genomes detected by conventional molecular methods originates from non-infectious particles. However, PMAxx™-based approaches do not provide a direct measurement of infectivity, although multiple studies have demonstrated a correlation between PMA reduction and cell culture for RNA viruses (e.g., enteroviruses, noroviruses, and SARS-CoV-2), supporting the use of photoactivatable dyes as a proxy for viral structural integrity and potential infectivity [13,14,25]. This study extends the applicability of this approach to WNV, for which information linking molecular detection to infectivity in clinical samples remains limited.

Initially, the method was tested for efficacy against WNV by preparing dilutions of the virus in cell culture medium without FBS; treatment of these dilutions with PMAxx resulted in an average reduction of 74.6%. A dilution of the virus was also inactivated using UV light to verify that PMAxx prevented the amplification of free genomes or those present only within damaged viral particles. Indeed, it is known that UV light inactivates genomes but does not damage viral particles [26]. Therefore, as shown in Figure 1B, this result can be observed: UV light significantly reduces the viral load, and this viral load damaged by UV rays is likely surrounded by intact viral particles that PMAxx cannot reach; indeed, the viral load following UV exposure and subsequent treatment with PMAxx is not significantly different from that treated with UV alone.

Furthermore, to verify whether a different type of matrix might influence the efficacy of PMAxx, we carried out serial dilutions of WNV in urine and plasma samples, where the average reduction rate was 73.5% and 71.0%, respectively, similar to that obtained for viral dilutions in cell culture medium, which was 74.6%. This demonstrates that the matrices used in this study do not affect the correct functioning of PMAxx. To confirm the results obtained, it was also verified that the use of non-activated PMAxx did not reduce the WNV viral load in the samples, thereby demonstrating that the process was carried out correctly.

When considering clinical samples, Spearman correlation test confirmed a significant association between NT values and the results of PMAxx treatment in all three groups, but linear regression analysis showed a significant association when all samples were considered together or for WNF, and no significant association was found for WNND. Taken together, these results probably highlight that there is a linear trend among the samples that did and did not undergo PMAxx treatment, although it is not well-defined. This is probably because the outcome observed after PMAxx treatment does not depend on the initial viral load detected in the untreated sample, but rather only on the intact viral particles present in the sample. This result is supported by the data shown in Figure 7. In one plasma sample, a comparable WNV copy number was detected both before and after PMAxx treatment, whereas in a urine sample, a high viral copy number markedly decreased after treatment to only 115 copies/mL. Accordingly, the former sample was successfully isolated, while the latter was not. This outcome is probably attributable to the well-known sensitivity limits of viral isolation. The other samples shown in Figure 7C,E tested negative for WNV after PMAxx treatment, and this result is consistent with the lack of viral isolation.

Analyzing all clinical samples together, PMAxx™ treatment resulted in a median difference of 1.0 Log copies/mL, indicating that one order of magnitude of detected viral RNA may not represent potentially infectious virus. This finding is consistent with previous studies showing that photoactivatable dyes selectively suppress amplification from viral particles with damaged envelopes or capsids, thereby improving the biological relevance of molecular quantification [11,12,13,14,25].

Plasma samples showed 0.4 Log copies/mL as the median difference, suggesting that a large fraction of circulating viral RNA is associated with intact viral particles. In contrast, urine samples exhibited 1.8 Log copies/mL as the median difference, indicating a higher proportion of RNA from structurally compromised virions or free RNA in this matrix with respect to plasma. This finding provides molecular support for previous observations that WNV RNA can persist in urine for prolonged periods without clear evidence of infectious viruses [23,27]. In addition, the high levels of intact viral particles in plasma could reinforce evidence of non-vectorial WNV transmission via blood transfusion and organ transplantation [28]; further studies on organ tissue samples would need to be conducted to identify the presence of intact viral particles.

Moreover, stratification by clinical symptoms further revealed that both WNF and WNND patients experienced significant reductions in viral RNA following PMAxx™ treatment, with comparable median differences across matrices. Notably, no statistically significant differences were observed between the two clinical groups. This suggests that clinical symptoms were not associated with a higher proportion of PMAxx™-resistant viral particles. Interestingly, urine samples from WNND patients showed the highest untreated viral loads, yet also demonstrated some of the largest PMAxx™-mediated reductions. This suggests that while urine remains a highly valuable specimen for increasing the diagnostic sensitivity of WNV detection [29], high RNA concentrations in urine do not necessarily correspond to sustained viral infectivity [7], reinforcing the notion that molecular positivity in this matrix should be interpreted with caution when used to infer viral infectivity or transmission potential.

## 4. Materials and Methods

### 4.1. Clinical Specimens

From July to September 2025, 55 biological specimens (20 urine and 35 plasma samples) were collected for diagnostic purposes from 41 patients, 17 (41.5%) females and 24 (58.5%) males. Patients had a median age of 69 years (interquartile range (IQR): 28.5 years); 20 patients had WNND, and the other 21 had WNF. WNND classification was based on clinical and laboratory criteria and when patients presented with neurological symptoms, in combination with the detection of anti-WNV IgM in the cerebrospinal fluid (CSF) sample. The median time to symptom onset for WNND was 5 days (IQR: 3.5), while for WNF, it was 6 days (IQR: 3). According to the Mann–Whitney test, there were no significant differences in symptom onset between the two groups. Specimens were sent to the Laboratory of Virology of National Institute for Infectious Diseases Lazzaro Spallanzani—IRCCS in Rome, as the Regional Reference Laboratory for arboviral infections, for diagnostic and surveillance purposes. Once samples were tested for the presence of WNV, they were anonymized (retaining only information on sex, age, and type of symptoms) and stored at −80 °C.

### 4.2. PMAxx Treatment

**Figure 8:**
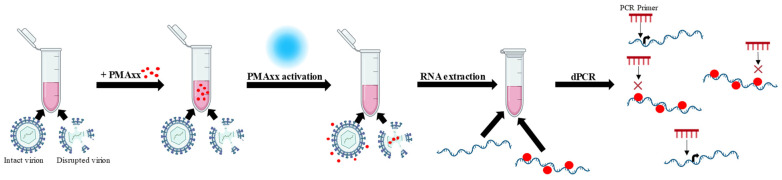
Mechanism of action of the PMAxx.

PMAxx treatment was carried out on 100 μL biological specimens, using 50 μM of PMAxx™ Dye (Biotium, San Francisco, CA, USA [30]). The PMA-Lite™ 2.0 LED Photolysis Device [30] was used to photoactivate PMAxx™ for 30 min, according to the manufacturer’s instructions [30]. Notably, 50 µM was the most used concentration for PMAxx [31,32], and, in a previous study [13], it was found that 50 μM PMAxx™ was optimal, as increasing it to 200 μM did not change the number of copies detected (Figure 8).

### 4.3. WNV Quantification

WNV RNA was extracted from PMAxx-treated and not-treated (NT) samples by using the QIAamp Viral RNA Mini Kit (Qiagen, Milano, Italy), according to the manufacturer’s instructions [33]. The dPCR reaction was performed to effectively estimate the viral RNA copy number using the QIAcuity One Platform System (Qiagen, Milano, Italy), 5-plex and QIAcuity Nanoplate, microfluidic dPCR plates and setup as follows: 10 μL of 4× QIAcuity Probe PCR Kit (Qiagen, Milano, Italy), 0.5/0.15 μM of the primers/probes mix for WNV [34], 10 μL of RNA, and RNase-free water were mixed to reach a final reaction volume of 40 μL. Data were analyzed using the QIAcuity Suite Software V1.1.3 193. For NT samples, the same sample volume used for PMAxx-treated specimens was processed.

### 4.4. WNV Isolate and UV Inactivation

This study used WNV isolate (WNV2-INMI-1-2025; GenBank: PX024392.1) with a titer of 10^8.82^ TCID_50_/mL measured by the Reed and Muench method [35] on Vero E6 cells. The isolate was diluted in cell culture medium, plasma matrix, and urine matrix up to 10^0.82^ TCID50/mL for the analysis with PMAxx.

To verify the PMAxx activity on inactivated virus, serial dilutions of WNV were exposed to UV light for 30 min in ice.

### 4.5. WNV Isolation from Clinical Samples

Viral isolation was performed in a BSL-3 laboratory using Vero E6 cells, in accordance with previously described procedures [12,13]. Briefly, samples were diluted in MEM supplemented with antibiotics and antimycotics and incubated at room temperature for 30 min. The treated suspensions were then inoculated onto Vero E6 cell monolayers and incubated for 1 h at 37 °C in the presence of 5% CO_2_. After incubation, the inoculum was removed and replaced with MEM containing 2% fetal bovine serum and the antibiotic–antimycotic mixture. Cells were subsequently monitored for cytopathic effects using light microscopy (Nikon Europe B.V., Amstelveen, The Netherlands).

### 4.6. Statistical Analysis

Data management and analyses (median, IQR, Wilcoxon test, Kolmogorov–Smirnov test, Mann–Whitney test, Spearman correlation test, and linear regression test) were performed using GraphPad Prism version 9.3.1 (GraphPad Software, Boston, MA, USA [36]). Wilcoxon test was used to evaluate whether the dPCR results of NT and PMAxx-treated samples were statistically different. Kolmogorov–Smirnov test was used to verify whether there were significant differences in terms of reduction due to PMAxx between WNND and WNF. Spearman correlation test and linear regression analysis were performed to evaluate the correlation between NT and the difference (Δ) between NT and PMAxx-treated samples. For graphical representation and statistical analysis, an arbitrary value of one copy/mL was assigned to negative samples.

## 5. Conclusions

The PMAxx™-dPCR workflow represents a promising strategy to enhance the biological relevance of molecular WNV diagnostics. By improving the discrimination between residual viral RNA and RNA associated with potentially infectious particles, this approach addresses a critical gap in WNV diagnostics. Although further studies integrating molecular, virological, and clinical data are needed to fully validate its clinical utility, the present findings suggest that PMAxx™-dPCR could become a valuable adjunct to conventional molecular and culture assays for WNV detection.

## Data Availability

The original contributions presented in the study are included in the article; further inquiries can be directed to the corresponding author.
